# A High-Throughput, Arbitrary-Waveform, MPI Spectrometer and Relaxometer for Comprehensive Magnetic Particle Optimization and Characterization

**DOI:** 10.1038/srep34180

**Published:** 2016-09-30

**Authors:** Zhi Wei Tay, Patrick W. Goodwill, Daniel W. Hensley, Laura A. Taylor, Bo Zheng, Steven M. Conolly

**Affiliations:** 1 Department of Bioengineering, 340 Hearst Memorial Mining Building, University of California, Berkeley, Berkeley, CA, USA; 2 Magnetic Insight, Inc. 980 Atlantic Avenue Suite102 Alameda, CA 9450, USA; 3 Department of Electrical Engineering and Computer Sciences, 253 Cory Hall, University of California, Berkeley, Berkeley, CA, USA.

## Abstract

Magnetic Particle Imaging (MPI) is a promising new tracer modality with zero attenuation deep in tissue, high contrast and sensitivity, and an excellent safety profile. However, the spatial resolution of MPI is limited to around 1 mm currently and urgently needs to be improved for clinical applications such as angiography and brain perfusion. Although MPI resolution is highly dependent on tracer characteristics and the drive waveforms, optimization is limited to a small subset of possible excitation strategies by current MPI hardware that only does sinusoidal drive waveforms at very few frequencies. To enable a more comprehensive and rapid optimization of drive waveforms for multiple metrics like resolution and signal strength simultaneously, we demonstrate the first untuned MPI spectrometer/relaxometer with unprecedented 400 kHz excitation bandwidth and capable of high-throughput acquisition of harmonic spectra (100 different drive-field frequencies in only 500 ms). It is also capable of arbitrary drive-field waveforms which have not been experimentally evaluated in MPI to date. Its high-throughput capability, frequency-agility and tabletop size makes this Arbitrary Waveform Relaxometer/Spectrometer (AWR) a convenient yet powerfully flexible tool for nanoparticle experts seeking to characterize magnetic particles and optimize MPI drive waveforms for *in vitro* biosensing and *in vivo* imaging with MPI.

Magnetic Particle Imaging (MPI), a new tracer-based molecular imaging technique, directly detects and quantifies the intense magnetization of superparamagnetic iron oxide (SPIO) tracers in the body. This unique contrast mechanism, combined with its use of low-frequency magnetic fields and clinically safe magnetic tracers, enables MPI to produce clinical-grade images with zero tissue signal attenuation, high contrast-to-noise ratio, and high sensitivity, all with a better safety profile than nuclear medicine^1^,^2^,^3^. Like MRI, it is bound by Magnetostimulation and Specific Absorption Rate (SAR) safety limitations^4^,^5^. MPI is best compared to gold-standard tracer imaging techniques, such as nuclear medicine (PET, SPECT and scintigraphy), but without the limitations of radiation safety or radionuclide half-life for longitudinal imaging (Fig. 1). As such, MPI shows excellent promise for clinical applications such as angiography, stem cell tracking^6^,^7^,^8^, brain perfusion, lung ventilation^9^, cancer imaging and hyperthermia^10^,^11^. A representative MPI *in-vivo* image of stem cell tracking demonstrating excellent contrast, zero tissue signal, high sensitivity and capability for longitudinal imaging is shown in Fig. 1.

Beyond these clinical applications, MPI may also be useful for biosensing applications both *in vitro* and *in vivo*. Recently, MPI scanners and spectrometers have been used for probing tracer microenvironment to determine the dose of magnetic tracer loading in cells^7^,^8^,^12^,^13^, to non-invasively assess cell vitality^14^, and to generate colorized MPI contrast based on tracer microenvironment differences^15^,^16^. Furthermore, recent work by Kuhlmann *et al*.^17^ have shown progress in detecting SPIO mobility for binding contrast via the use of two frequencies to decouple the concentration and SPIO relaxation information. These applications may be crucial for future clinical applications, such as determining the vitality of cell-based therapeutics (combined with stem cell tracking studies such as Fig. 1) and tracking the biodistribution and clinical efficacy of magnetically-tagged immunotherapies.

The spatial resolution of native MPI images, limited by scanner hardware and the magnetic behavior of MPI tracers at around 1 mm currently, is a prime target for improvement. This is an important limitation especially when MPI is used for visualization of anatomy. For example, in Fig. 1, the shape of the spleen is not well resolved. Most of the effort to-date has been directed to designing better MPI tracers^18^,^19^,^20^,^21^,^22^,^23^,^24^,^25^ and improved scanner hardware with higher magnetic gradients^26^,^27^,^28^. Recent literature have shown that the drive waveform affects MPI spatial resolution^29^,^30^,^31^,^32^,^33^,^34^,^35^. Unfortunately, current MPI hardware is limited to only sinusoids at a few discrete frequencies^2^,^3^,^26^,^27^,^28^,^36^,^37^,^38^,^39^,^40^,^41^,^42^. Multiple simultaneous frequencies (Philips-Bruker 3D Fast MPI demonstrator scanner^15^) is limited to a narrow range (24.51 kHz, 25.25 kHz, and 26.04 kHz). Discrete drive frequencies at 10, 25, 50 and 100 kHz was shown by Kuhlmann *et al*.^29^, although denser frequency sampling is helpful in optimizing for both spatial resolution and signal strength. Also, the use of two frequencies to decouple signal intensity and SPIO mobility information by Kuhlmann *et al*.^17^ suggests that denser frequency sampling could further optimize color MPI contrast. Furthermore, the use of square waves in the ferrofluid literature^43^,^44^ and constant velocity scanning (same scanning trajectory as triangular drive wave) by Vogel *et al*.^45^ showing benefits of simplified reconstruction suggest that various unexplored arbitrary drive-field waveforms could be useful in MPI.

All of these recent studies suggest that a high-throughput, frequency-flexible, arbitrary-waveform MPS device will be of significant interest to the MPI community for optimizing drive waveforms for spatial resolution, signal strength and contrast. In order to build hardware capable of arbitrary drive frequencies, the main technical challenges are to generate MPI drive fields with a wide bandwidth while reducing direct feedthrough and reactive power. Previous MPI hardware have been unable to meet this requirement as bandstop filters are used to deal with feedthrough and/or tuned circuit elements are used for reactive power handling^46^,^47^,^48^,^49^,^50^,^51^. Figure 2b shows a typical circuit design in conventional MPI hardware. Thus, prior work has been limited to a few discrete drive frequencies. Changing frequencies require time-consuming switching of tuning capacitors^47^,^48^, although some frequency-tunability within a small bandwidth (19–25 kHz) is possible^47^.

In this paper, we present a novel Magnetic Particle Spectrometer that is uniquely capable of high-throughput and comprehensive optimization of the MPI drive waveform. We greatly expand the optimizable parameter space with unprecedented frequency agility across DC – 400 kHz (Fig. 3c) and capability for non-conventional drive waveforms. We also obtain harmonic spectra at 100 different drive frequencies (16–115 kHz) within a single 500 ms acquisition (Fig. 4a), demonstrating more than 100-fold improvement in throughput due to time savings from instantaneous frequency switching. Because we have validated against an MPI imager the accuracy of spatial resolution measurements from this device (Fig. 5b), this hardware allowed for the first time experimental evaluation of MPI performance under arbitrary drive waveforms (Figs 6 and 7). Thus, the AWR’s tabletop size without need for dedicated facilities poses it as a convenient yet powerfully flexible tool for nanoparticle experts looking to characterize magnetic nanoparticles as well as MPI systems designers looking to optimize drive waveforms for MPI resolution and signal strength.

## Design and Methods

### Working Principles of Magnetic Particle Imagers

The basis of MPI is the non-linear magnetization response of superparamagnetic iron oxide (SPIO) tracers. Magnetic gradient fields are applied in order to magnetically saturate SPIOs in all spatial regions other than the field-free-region (FFR) of the gradient field. The FFR may be shaped as a line (FFL) or as a point (FFP). The FFR is then shifted rapidly (at frequencies of 10–25 kHz) by a drive coil and the corresponding induced signal from the magnetization response of SPIOs spatially located at the FFR is picked up by an inductive receive coil. The combination of the gradient and drive coils shift the FFR across the entire field of view and allows reconstruction of the image from the voltage time series. Reconstruction can be done in the frequency domain by obtaining a system function and then solving the inverse problem of the spectral image data^52^. This can also be done in the time domain by mapping the time domain signal to a spatial grid through knowledge of the instantaneous FFR location^2^. In the literature the former approach is often referred to as system matrix reconstruction while the latter is referred to as x-space reconstruction^2^,^30^,^41^,^42^,^53^,^54^,^55^.

High quality MPI requires a large magnetic moment and a steep dynamic magnetization curve or M(H) response. This yields a sharp Langevin curve. In the frequency domain, this implies higher amplitudes for all harmonics and a gentle slope of the harmonic decay envelope^29^. In x-space reconstruction, this corresponds to a tall and narrow point-spread function (PSF) where spatial resolution is quantified with the Houston criterion^56^. In this paper, we will use x-space reconstruction. Similar conclusions may be obtained with harmonic or system-matrix reconstruction.

As such, the non-normalized peak height of the x-space PSF and its FWHM are thus key parameters for characterizing tracer performance in MPI. Deconvolution could be applied in post processing to improve resolution as demonstrated in prior MPI work^57^,^58^,^59^,^60^.

### Design of the Arbitrary Waveform Spectrometer/Relaxometer

To measure these key MPI performance metrics, an MPI relaxometer or spectrometer is necessary. Magnetic Particle Spectrometers (MPS) were first developed in 2009 by Biederer *et al*.^39^ and 2010 by Wawrzik *et al*.^37^ and measures the SPIO response to a pure tone drive field. Because no gradients are used, the response is that of a SPIO point source located at the FFP (or another location if a homogeneous biasing field is applied). Other magnetization characterization devices are typically inadequate in obtaining these MPI performance metrics because the field amplitudes needed to reach the non-linear regions of the tracer magnetization curve are not achieved in AC susceptometry^29^. Furthermore, static measurements such as Vibrating Sample Magnetometry (VSM) are inaccurate in predicting MPI performance at 10–25 kHz because of temporal relaxation effects^34^. Figure 3 compares the capabilities of an MPI relaxometer and other characterization tools and shows that AC susceptometry and Vibrating Sample Magnetometry (VSM) only cover a small fraction of the MPI-relevant parameter space.

To achieve the high magnetic field amplitudes needed to excite the MPI tracers, reducing reactive power required by the drive coil is necessary. While prior work uses tuned circuit elements to achieve this^46^,^47^ (Fig. 2), arbitrary drive waveforms need a wideband method to reduce reactive power. Miniaturizing the MPI drive coil^61^ is one way to do this.

All transmit and receive coils were wound on custom-designed, miniature 3D-printed bobbins or scaffolds depicted in Fig. 2. Our solenoidal drive coil has 18 turns of 175/44 served litz copper wire with 8.4 mm inner diameter and 1.9 cm length. The detector (receive) coil fits inside the drive coil and has 20 turns of 100/44 served litz copper wire shaped to the sample holder (0.2 mL PCR tube) dimensions. A duplicate set of detector coil is spaced ~4 mm away and connected in series to the primary for gradiometric cancellation. The net drive coil inductance and efficiency are 2.5 *μ*H and 1.06 mT/A respectively. This results in significantly lower reactive power compared to prior work with 75 *μ*H inductance and 0.77 mT/A drive coil efficiency^47^. Thus, the AWR is capable of wideband excitation at any frequency between *DC* – 400 kHz and at field amplitudes of up to 86 mTpp (Fig. 3). The limit of 400 kHz is because magnetic relaxation times^62^ approach the drive wave period causing poor resolution and SAR safety limits are <1 mTpp beyond 400 kHz^5^.

The spatially homogenous drive field is generated by an untuned solenoid powered by a DC-coupled power amplifier (AE Techron 7224) with pulsed power of up to 1.2 kW. Drive field inhomogeneity within the sample volume of 25 *μ*L is <1%. While the small dimensions reduces sample capacity to 25 *μ*L, SNR per unit volume is improved^63^, and sufficient SNR (>10) even for cell labeling studies (~20 *μ*g/mL)^12^ is achieved. To match the load to the AE Techron 7224 optimal load range, a 1.0 Ω heatsink-mounted non-inductive resistor (LPS800 thick film resistor, Vishay Sfernice) was added in series to the transmit coil (Fig. 2b).

The next technical challenge is to deal with direct feedthrough interference which is typically many orders of magnitude higher than the SPIO signal^47^. For single tone excitation, the signal lost when filtering out the first harmonic can be recovered by a continuity algorithm^53^ since first harmonic information lost corresponds to a local DC offset. Therefore prior relaxometers using monotonal excitation have relied on analog bandstop filters (Fig. 2) to remove the excitation feedthrough^30^.

Unfortunately, in an arbitrary drive waveform context, the excitation feedthrough is wideband and analog bandstop filtering across such a wideband would likely render recovery of the tracer signal impossible. Therefore, gradiometric (wideband) attenuation of the direct feedthrough is the only practical method to reduce field feedthrough interference from tracer signal. A duplicate setup as cancellation unit idea^46^,^47^ perpendicular Tx and Rx^64^ idea, and three-section gradiometer^65^,^66^ have been proposed. We chose a two-section gradiometer design that is conceptually similar to first idea but with implementation of a novel mechanical design to adjust cancellation signal amplitude. This improved our wideband analog gradiometric attenuation of direct feedthrough to around −67 dB (−67 dB at 10 kHz and −63 dB at 400 kHz) as shown in Fig. 3.

After analog gradiometric attenuation, the received SPIO response is (analog) amplified by the SR560 low-noise preamplifier (Stanford Research Systems, USA) at 1 MHz bandwidth or the NF5307 differential amplifier (NF Corporation, Japan) at 10 MHz bandwidth. The amplified signal is then digitized at 10 MSPS by a 12-bit ADC (National Instruments PCI-6115, Austin, TX, USA). A baseline subtraction (background correction) is then performed digitally to further reduce residual feedthrough interference. To expand the field of view, we use a bias coil (Fig. 2) that produces up to ±120 mT homogeneous field when driven by a current-controlled amplifier (AE Techron LVC5050, Elkhart, IN, USA). The system is controlled using custom software written in MATLAB (Mathworks MATLAB, Natick, MA, USA).

### Nanoparticle Tracers

Aqueous suspensions of Resovist™ superparamagnetic iron oxide particles (Bayer Healthcare, Germany; 0.5 mmol Fe/mL) were used. Resovist is widely used in MRI^67^,^68^,^69^ and in MPI^29^,^30^,^31^,^34^,^70^,^71^. Although Resovist is known to have a very heterogeneous nature, the representative core diameter is ~17 ± 4 nm^34^. Unless otherwise stated, all experimental measurements employed 25 *μ*L of Resovist diluted to 5 mg Fe/mL.

Senior Scientific Precision MRX™ SPIOs (Azano Biotech, Albuquerque, NM, USA) with carboxylic acid coated outer shell and 32.1 nm core diameter was also used. Unless otherwise stated, all experimental measurements employed 25 *μ*L SPIOs at 5 mg Fe/mL.

### Animal Procedures

**Figure 1:**
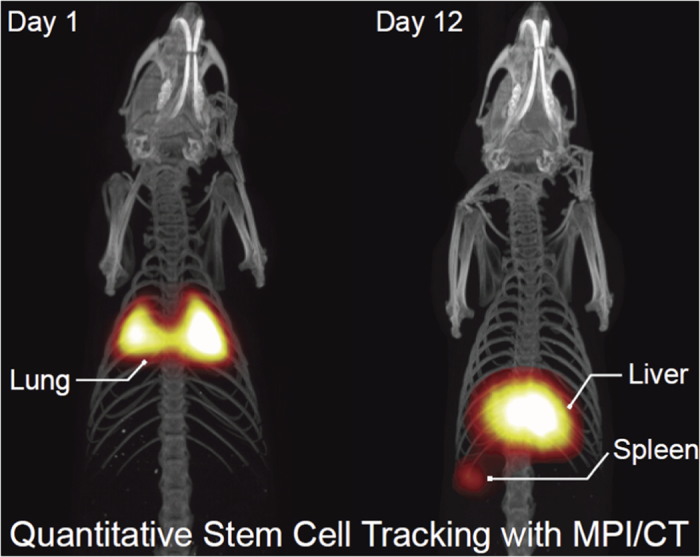
A representative MPI image showing visualization of the biodistribution of magnetic particle labeled stem cells after they were injected into the tail vein of a rat. This image demonstrates the high sensitivity and contrast of MPI and the capability to perform long-term, *in vivo* biodistribution studies without the exponential loss of signal typical in nuclear medicine studies. However, as shown by the image of the lung, liver and spleen in Fig. 1, the spatial resolution of MPI is limited and a prime target for improvement. We demonstrate in this paper that the AWR enables high-throughput drive waveform optimization for better MPI resolution.

All animal procedures were approved by the Animal Care and Use Committee at UC Berkeley and carried out in accordance with the National Research Council’s Guide for the Care and Use of Laboratory Animals. For Fig. 1, immunocompetent 7-week old female Fischer 344 rats weighing approximately 130 g were imaged with MPI. Animals were fed on a diet of Teklad Rodent Diet 2018 (Harlan, Indianapolis, IL) ad libitum. The rats (2 groups of n = 3) received tail vein injections of 5 × 10^6^ to 8 × 10^6^ Resovist-labeled hMSCs in 1 mL PBS. The hMSCs used were labeled with 40 *μ*g Fe/mL of Resovist in cell culture solution using methodology similar to previous studies^72^. A custom animal bed was used to support the animal under isoflurane anesthesia (2%, 1.5 L/min) within a MPI scans used a 4 × 3.75 × 10 cm FOV and a 9 minute acquisition. Although MPI scans have been performed *in vivo* on live animals^8^, for Fig. 1 the animals were sacrificed using isoflurane overdose for postmortem co-registered MPI and CT imaging at 1 day and 12 days post-injection. CT images were acquired (RS9-80 CT, 25 min acquisition, 184 *μ*m isotropic resolution) as an anatomic reference and the resultant MPI-CT 3D images were co-registered visually using SPIO-glass fiducial markers with Osirix Imaging Software (Pixmeo SARL, Switzerland).

### MPI Tracer Characterization using Sinusoidal Drive Waveform

The AWR measures the PSF of the SPIO sample, from which we can measure signal strength, spatial resolution and relaxation time constant^34^. Since it is not an MPI scanner, there are no gradient fields and the PSF is reported in units of magnetic field (mT). This can be easily translated to spatial distance (mm) by dividing by the MPI scanner gradient. The AWR drive coil produces a sinusoidal magnetic drive field at frequencies between 1–400 kHz with 1–43 mT peak amplitude. The bias coil simultaneously applies a linear ramp from 40 mT to −40 mT in 250 ms to ensure that the entire magnetic behavior of the sample, including the saturation regions, is interrogated. To reconstruct a PSF, we grid the velocity-compensated instantaneous induced voltage signal from our receive pick-up coil to the bins of a magnetic field grid via interpolation from the instantaneous applied field magnitude to the grid. We only use data corresponding to the center 10% of each half-period bracketing the zero-crossing for higher SNR. As the first harmonic is kept, DC recovery^53^ is not applied to each partial FOV or imaging station^34^,^53^,^73^,^74^,^75^. Overlapping points are averaged within each 0.1 mT wide bin to form the resultant PSF with edges set to zero. Positive and negative velocity scans are reconstructed separately.

Theoretical x-space PSFs were computed using MATLAB software (Mathworks MATLAB, Natick, MA). To model the effect of relaxation with any arbitrary transmit pulse sequence, we use the MPI Debye model for magnetic relaxation validated in Croft *et al*.^30^,^34^ with the specific equation:


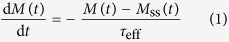


where *M*(*t*) is the instantaneous magnetization as a function of space, *M*_ss_(*t*) is the steady-state (Langevin) magnetization, and *τ*_eff_ is an effective, phenomenological magnetic relaxation time constant. Since no gradient is present, *M* and *M*_ss_ have no spatial dependence.

The exact pulse sequence used in the AWR was used for simulations. The inductive signal for a point source under the AWR pulse sequence was calculated by equation 1 under a finite difference method at 10 MHz sampling rate. This is equal to the AWR analog sampling rate and sufficient to capture the dynamics of relaxation times of more than 0.2 *μ*s. As with experimental data, the simulated data was reconstructed using the gridding procedure outlined above. To validate our AWR hardware, we replicated the sinusoidal excitation parameters of 30 mT and 9.3 kHz used in prior work^34^. For theoretical PSF modeling we used the validated relaxation time constant of 2.3 *μ*s and Resovist parameters of 17 nm mean core size with 4 nm standard deviation from prior work^34^. We then compared the PSF from AWR data to the theoretically calculated PSF.

To further verify the accuracy of spatial resolution predictions from our novel relaxometer (AWR), we compare resolution predictions of the AWR to measured *image* resolution in our Berkeley MPI imager (23 kHz and varying field strengths). The imaging scanner has been validated in our previous publications^34^,^40^,^42^. We use the image resolution values of the scanner from prior work^34^ and compare them to the PSF FWHM from our AWR data by dividing by the scanner gradient (3.5 T/m). To ensure an accurate comparison, we used the same frequency (23 kHz) and field strengths that were used to obtain the image resolution values in Croft *et al*.^34^. To account for the finite size of the phantoms used in the MPI imager, the AWR-measured PSF was convolved with the dimensions of the phantom in the measurement direction (1.0 mm diameter) before measurement of FWHM. The coefficient of determination (*R*^2^) between the imager-measured FWHM and AWR-measured FWHM was then calculated.

### Novel Implementation of Arbitrary Drive Waveforms

To demonstrate frequency-agility, we implemented on the AWR a linear chirp across DC – 400 kHz (amplitude 10 mT). We also implemented a composite arbitrary waveform composed of four contiguous parts: (1) triangular wave at 10 kHz and 25 mT amplitude (2) linear chirp running from 10 kHz to 50 kHz at 25 mT amplitude (3) ramp running from −25 mT to 25 mT over 0.2 ms (4) composite waveform made up by adding sine waves of 8.3 mT amplitude at 5, 7 and 12 kHz. All drive waveforms were verified by the real-time current monitor on the AE Techron 7224 power amplifier. The real-time SPIO response to the arbitrary waveform was recorded for Resovist and Senior Scientific 32 nm SPIOs and plotted on the same graph. The SPIO signal traces are normalized for better visibility.

To measure the triangular wave PSF, the entire acquisition and reconstruction uses the same sine wave protocol described in the previous section but with the drive waveform changed to that of a triangular wave.

## Results

### Validation of the Accuracy of the Arbitrary Waveform Relaxometer

To validate the AWR, we compare data taken from the AWR with theory, prior work and imaging results. Figure 5a shows that the reconstructed PSF from AWR data closely matches the PSF calculated from theory^34^. Figure 5b shows that the predicted resolution from the AWR closely matches (*R*^2^ = 0.972) the measured resolution from scanner *imaging results*, demonstrating that the AWR can accurately predict *imaging* spatial resolution in MPI. This verification allows us to use the AWR to predict MPI performance with arbitrary drive waveforms that cannot be performed on any current MPI scanner to-date. Hence, the AWR is a powerful investigation tool to evaluate the pros and cons of arbitrary drive waveforms for MPI before scaling up to animal-size scanning.

### Limit of Detection of 20 ng Fe

Different amounts of Resovist was diluted in DI water to 25 *μ*L volume and measured for 6.25 seconds. The peak amplitude of the reconstructed PSF is linear with the amount of iron (Fig. 5), demonstrating the AWR is quantitative for SPIO amount. We observed the limit of detection (SNR = 1) to be ~20 ng, showing more than 10-fold improvement from prior relaxometers^48^,^76^.

The improved sensitivity is likely due to these factors: (1) The small size of the receive coil increases SNR per unit volume^63^. (2) improvements in gradiometer performance (Fig. 3) shifts the ADC dynamic range lower to capture weaker SPIO signals. (3) The signal at the first harmonic is kept and not discarded unlike prior relaxometers with bandstop filters at the first harmonic.

### Automated High-Throughput Characterization of Nanoparticle Tracers

Because the AWR does not rely on the tuned circuit elements used in all prior work, it is capable of a continuous and wide range of frequencies and thus well positioned to conduct digitally automated, high-throughput studies of drive waveform parameters. In prior work, changing drive frequencies typically required time-consuming manual switching of tuned circuit elements. Even if this process could be automated, having tuned circuit elements limits the available frequencies to discrete values and precludes arbitrary waveforms.

To demonstrate potential for automated and high-throughput studies, in Fig. 4 we acquire the Fourier spectra of Resovist at 100 different drive-field frequencies (sinusoidal drive-field) that was automatically taken in a single acquisition (total acquisition time of 500 ms). There is no manual hands-on requirement except for an initial sample insertion of the Resovist probe. With automated frequency switching and good SNR with only 5 ms per drive-field frequency, the AWR is more than 100-fold faster than prior work^30^,^48^,^55^ requiring multiple capacitor exchanges to cover the same frequency range. The main time savings come from the rapid, capacitor-free frequency switching. Thus, measurement time can be increased above 5 ms if more SNR is desired. This demonstrates that the AWR can be automated to rapidly investigate the entire parameter space of drive frequency and amplitude for one tracer. Although not shown here, this could be easily extended to the parameter of arbitrary waveform shape as well.

In addition, we demonstrate optimization of drive waveforms for better spatial resolution in Fig. 4c that plots FWHM resolution of Resovist against 33 sets of drive parameters (frequency and amplitude). Crucially, this dataset was obtained from a *single* AWR acquisition, demonstrating capability for high-throughput optimization. Because lower drive amplitudes were investigated here, the biasing coil was used to extend the applied field and limits throughput to the biasing coil slew rate. As a result, a longer acquisition time of 8.25 s is required. The data shows a trend of worsening resolution with larger drive-field frequency and/or amplitude. This matches the conclusions from prior work^29^,^34^ and validates the accuracy of the AWR. For example, our data at 25 kHz and for amplitudes of 8.5, 17, 25 mT, have FWHM of 7.0, 9.4, 10.5 mT respectively. This closely matches the values of 7.0, 9.1, 10.3 mT from Croft *et al*.^34^.

Furthermore, as a result of denser sampling of the drive frequencies, we are able to optimize the trade-off between spatial resolution and signal strength. For example, Fig. 4d shows a non-obvious drive waveform that gives the best resolution while having the same signal strength as a conventional MPI waveform.

### Arbitrary Drive Waveforms

The AWR is capable of a linear chirp drive waveform from 1 kHz to 400 kHz (Fig. 6) within 500 ms, demonstrating the frequency-flexibility of the AWR. The chirp waveform amplitude starts to drop above 200 kHz due to power amplifier voltage slew rate limitations (Fig. 3). Furthermore, the AWR is capable of arbitrary drive waveforms as demonstrated by a waveform made up of contiguous arbitrary waveform parts: (1) triangular wave (2) linear chirp (3) ramp and (4) composite waveform (tri-tone). All drive waveform shapes are validated by the internal current monitor of the AE Techron 7224.

The response of two different SPIOs (Resovist and Senior Scientific 32 nm) are recorded. Visibly different responses to the arbitrary waveform in can be observed between Resovist and Senior Scientific 32 nm SPIOs because the larger 32 nm magnetic core SPIOs are known to have Brownian-dominant relaxation mechanisms and thus have a significantly delayed reaction to the applied field. These results suggest that the AWR is a good platform to test if non-sinusoidal waveforms may provide even better contrast between different SPIOs for colorized MPI imaging first pioneered by Rahmer *et al*.^15^.

### Wideband Feedthrough Attenuation

Because the analog feedthrough interference is no longer at a single frequency, bandstop filters cannot be used and the gradiometer becomes the only way to reduce analog feedthrough interference. In Fig. 3, we demonstrate that the gradiometer achieves wideband feedthrough attenuation of about −67 dB (−67 dB at 10 kHz and −63 dB at 400 kHz). We show similar limit of detection (SNR = 1) for a sine wave excitation (without bandstop filters) as that of a triangular wave excitation (Fig. 5), demonstrating that the combination of inductive decoupling and baseline subtraction is effective regardless of monotonal or wideband feedthrough interference.

### First Experimental Verification of x-space DC Recovery Algorithm

In prior work, feedthrough corruption of the first harmonic signal usually required analog bandstop filtering at the first harmonic. The lost first harmonic information is recoverable by a DC-recovery continuity algorithm during x-space reconstruction^42^,^53^. While this approach is well-posed and robust, removing this x-space reconstruction step could improve SNR and speed up reconstruction time. The theory and experimental demonstration of LSI properties are shown in Lu *et al*.^53^, but due to prior relaxometer hardware limitations it was not possible to experimentally compare the DC recovery PSF to a PSF with first harmonic information retained.

**Figure 2:**
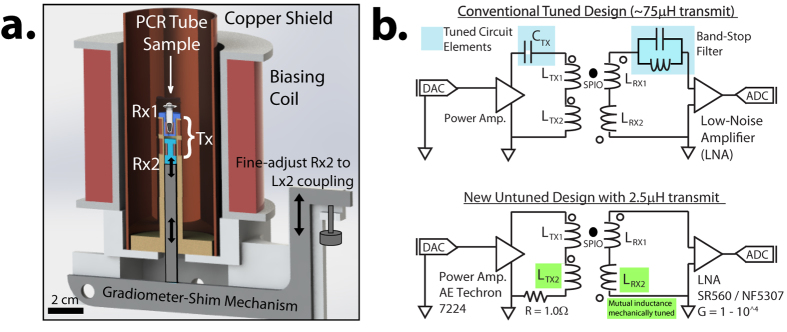
(**a**) To-scale Solidworks drawing™ of our arbitrary waveform relaxometer (AWR) accurately representing the actual physical device. It comprises a miniature drive coil (Tx) with a low net inductance of 2.5 *μ*H, two receive coils in gradiometric configuration (Rx1, Rx2), and a biasing coil to extend the applied field range. The gradiometer-shimming mechanism shifts Rx2 relative to Tx2 in increments of 22 *μ*m to fine-tune the Tx2-Rx2 coupling to match that of Tx1-Rx1, minimizing net Tx-Rx coupling. The concept of cancellation amplitude adjustment for inductive decoupling is not new^46^,^48^, but our novel mechanical implementation allows for in-bore facile precision “spatial-shimming” allowing for simultaneous feedback of gradiometer performance during adjustment. The in-bore adjustment is important because removing, adjusting then re-inserting the receive coil into the MPS setup may incur placement error and we have shown in Fig. 3a that even tens of microns can affect gradiometer performance. Prior hardware using inductive decoupling^46^,^47^,^48^ do not allow in-bore adjustment. (**b**) Prior sinusoidal MPI spectrometers/relaxometers requires capacitors in the transmit chain to reduce reactive power and/or a band-stop filter (BSF) in the receive chain to reduce feedthrough. However, arbitrary drive waveforms precludes the use of tuned circuit elements. Instead, the AWR’s novel untuned design relies on a very low coil inductance of 2.5 *μ*H coupled with a high coil efficiency of 1.06 mT/ampere for transmit power handling. An improved gradiometer is used for broadband feedthrough attenuation on the receive.

Our AWR is able to perform this first x-space experimental comparison due to the improvements in gradiometer design as outlined in Fig. 2 that obviates the use of bandstop filter on the receive chain. As a result, the AWR is able to reconstruct the PSF while keeping the first harmonic information. In Fig. 7 we show this *first experimental* comparison of the reconstructed PSFs using (1) x-space DC recovery method and (2) without DC recovery (first harmonic information was not filtered out). The results demonstrate that the DC recovery algorithm is robust and produces a nearly identical PSF as the PSF from retaining the first harmonic information.

However, because the DC recovery stitching operation of partial FOVs depends on an edge continuity condition, the noise at higher harmonics will be propagated when recovering the first harmonic. Our experiments show an estimated 4-fold SNR boost when retaining the first harmonic information at low iron content (<100 ng Fe).

### First Experimental Demonstration of Triangular Wave Drive Waveform

Triangular drive waveforms are useful as MPI drive waveforms because they imply fully linear motion of the MPI field-free-point (FFP across the field-of-view (FOV) in the presence of linear background magnetic gradients. The benefits of simplified reconstruction due to constant velocity have been shown in the work on Traveling Wave MPI by Vogel *et al*.^45^ where a dynamic linear gradient array (replacing both drive and background gradient) generates constant velocity motion of the FFP while using sinusoidal drive for each element. To assess the performance of the SPIO under a triangular drive waveform, we tested the same Resovist sample with a sinusoidal and triangular excitation. Both drive waves have exactly the same frequency and amplitude (9.3 kHz and 30 mT), differing only in their shape. The PSFs are nearly identical showing that a triangle wave excitation has a similar MPI performance as that of a sine wave (Fig. 7) but with simplified reconstruction (constant velocity).

## Discussion

**Figure 3:**
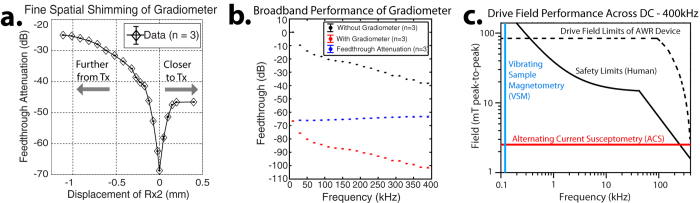
(**a**) Demonstration of improved gradiometer performance via fine “spatial-shimming” the cancellation coil (Rx2) location relative to the transmit coil (Tx). This has the effect of fine adjusting the Rx2-to-Tx coupling factor for improved inductive decoupling reaching up to −67 dB (10 kHz). (**b**) The gradiometer is capable of wideband feedthrough attenuation which is essential in the AWR’s wideband excitation context. (**c**) The very low drive coil inductance of 2.5 *μ*H coupled with high coil efficiency of 1.06 mT/ampere enables high field amplitudes across an unprecedented DC – 400 kHz despite not using resonant circuits for reactive power handling. When compared to the safe scanning limits for a human^5^, we see that the AWR’s unprecedented drive-field flexibility allows for testing of almost any drive waveform that would be used in a safe human scanning context, enabling comprehensive drive waveform optimization. In contrast, conventional VSM^77^ and AC Susceptometry^78^ are unable to cover the MPI-relevant parameter space. We limit our device design to below 400 kHz because near zero-field, the delay from magnetic relaxation is expected to be >2 *μ*s^62^, surpassing a half-period of the drive waveform and causing poor resolution.

The characteristics of the SPIO tracers used are critical to the imaging performance of MPI. While there are many tools available for analysis of these tracers such as Vibrating Sample Magnetometry, an MPI-specific tool is needed to measure the *imaging performance* of tracers. Therefore, the main role of a magnetic particle relaxometer is to rapidly and accurately characterize MPI tracer performance in a convenient fashion. Here, we have demonstrated that our relaxometer is able to accurately obtain the PSF of the tracer of a gold-standard particle Resovist™ and these measurements matches well with theory, prior work^34^, and most importantly, *imaging results* (Fig. 5). The actual measurement (without biasing coil) only takes 250–500 ms and even with computation, time writing data to disk, and 25 averages, it takes less than 30 seconds to measure a sample at a 100 drive frequencies (Fig. 3a). The table-top size of our AWR, the relative simplicity of construction (no tuned circuit elements), and the combination of speed and unprecedented frequency-flexibility make it a powerful and convenient tool for MPI researchers.

Up till recently, Magnetic Particle Spectrometers only used very few discrete frequencies and were mainly for particle characterization at conventional MPI drive waveforms. However, recent work in the MPI literature using Magnetic Particle Relaxometers/Spectrometers have demonstrated that both drive frequency and amplitude have a significant effect on MPI spatial resolution^29^,^30^,^31^,^32^,^33^,^34^. Furthermore, Vogel *et al*.^45^ showed through the work on Traveling Wave MPI the many compelling benefits of linear motion of FFP (achievable with triangular drive waveforms) due to the constant FFP velocity that simplifies the mathematics and facilitates image reconstruction. In addition, the use of two different frequencies to decouple signal intensity and SPIO mobility information as shown by Kuhlmann *et al*.^17^ demonstrates the importance of varying drive frequency for contrast in color MPI. All of these suggest that it is of significant interest for Magnetic Particle Spectrometers (MPS) to take on a new and additional role of optimizing the MPI drive waveform as well.

**Figure 4:**
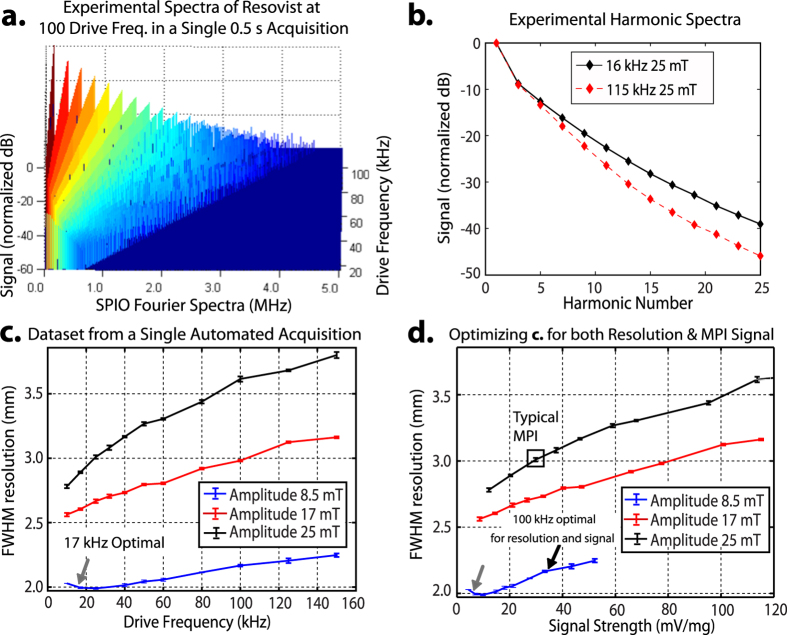
(**a**) High-throughput magnetic particle spectrometry is enabled by the lack of tuned circuit elements in the AWR. In a single automated acquisition (500 ms total time), we discretely sample 100 drive frequencies from 16 kHz to 115 kHz at 25 mT. The data from this single acquisition is shown as a stack plot of Resovist fourier spectra for 100 discrete drive frequencies (after background correction and removal of out-of-band signal). (**b**) Analysis of the spectra in Fig. 3a shows the expected steeper slope of harmonic decay with higher drive frequencies. This matches the findings of prior work^29^. A steeper slope implies a poorer modulation transfer function response leading to poorer spatial resolution. (**c**) From a single automated acquisition, 33 unique sets of drive parameters were tested (n = 3) on 125 *μ*g of Resovist. The optimal drive waveform (gray arrow) with best resolution is with a 17 kHz, 8.5 mT amplitude waveform. The FWHM resolution (mm) assumes a 3.5 T/m gradient. (**d**) The same dataset from part c is plotted for both spatial resolution and signal strength. While the lowest amplitude and frequency (gray arrow) gives the best spatial resolution, this is at a significant cost of almost 10-fold lower signal strength which has implications for MPI sensitivity. The high-throughput and denser sampling of frequency uniquely allows the AWR to better optimize for both spatial resolution and signal strength. This reveals the 100 kHz, 8.5 mT amplitude waveform (black arrow) which shows almost as good resolution improvement as the gray arrow while having *no loss in signal strength*, therefore having better overall MPI performance than the waveform obtained from simply optimizing for one parameter.

**Figure 5:**
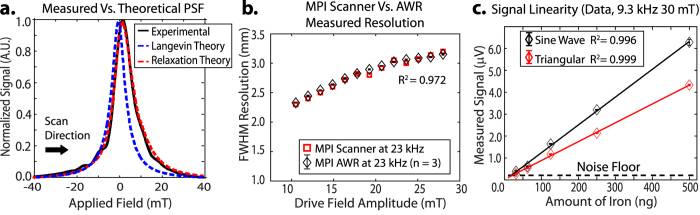
(**a**) Reconstructed PSF from data obtained by the AWR compares well to the theoretical expected PSF with magnetic relaxation of a first-order Debye model. The calculation used the same value of *τ* (2.3 *μ*s) validated in prior work^34^. (**b**) The FWHM of reconstructed PSFs from AWR data (n = 3) closely matches (*R*^2^ = 0.972) actual spatial resolution measured from MPI scanner *images* (3.5 T/m gradient 23 kHz)^34^. This shows the AWR can accurately predict for MPI tracer *imaging* performance. (**c**) The AWR signal (PSF peak amplitude) is linear with Resovist iron mass with sensitivity of 13.1 *μ*V/*μ*g for sine wave and 8.3 *μ*V/*μ*g for triangular wave before amplification. Three experimental repeats were taken per data point (n = 3). We estimate the detection limit (SNR = 1) to be ~20 ng and ~30 ng respectively with 6.25 s total acquisition time (25 averages). The AWR is thus a sensitive and quantitative sensor for magnetic particles. The difference in sensitivity between sine and triangular wave is due to differences in the waveform velocity at the zero-crossing point.

An ideal MPS hardware for comprehensive optimization of the drive waveform would be high-throughput, frequency-flexible across a wide range and also capable of arbitrary waveforms. Towards this goal, we consider that the 3D MPI platform from Bruker and Philips^73^ is capable of 3 simultaneous drive frequencies (excitation field) with programmable amplitude but the frequencies are very close (24.51 kHz, 25.25 kHz, and 26.04 kHz). The MPS platform from Kuhlmann *et al*.^29^ is capable of 4 discrete frequencies of 10, 25, 50 and 100 kHz, but it is of interest to more finely sample within this frequency range and beyond as shown by the non-obvious optimization results in Fig. 4d. To date, there has been no MPS hardware with the above-mentioned ideal specifications of full frequency-flexibility, high-throughput and arbitrary waveforms.

In response to this, we have designed our AWR to have unprecedented frequency-flexibility across DC-400 kHz (up to 86 mTpp), unprecedented arbitrary-waveform capability with orders of magnitude higher throughput than prior spectrometers. Although there may be no resonant effects expected from the SPIO tracer, due to the multitude of performance metrics to simultaneously optimize for, there is significant utility in high-throughput, dense sampling of the frequency parameter. We demonstrate this by revealing an optimum waveform of 100 kHz, 8.5 mT for Resovist that allows for significantly improved spatial resolution without compromising signal strength. In contrast, optimizing only for spatial resolution (lowest frequency and amplitude) incurs a significant 10-fold loss in signal strength (Fig. 4c) for marginal improvement in resolution.

**Figure 6:**
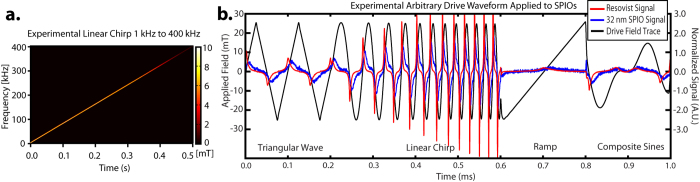
(**a**) Experimental data showing the linear chirp from 1–400 kHz produced by the AWR (spectrogram obtained from short-time Fourier Transform of AWR current monitor trace). Although the maximum slew rate of the power amplifier limits chirp amplitude beyond 200 kHz, this amplitude never falls below the SAR safety limits (Fig. 3) even at 400 kHz, thus covering the entire *safe* parameter space. (**b**) The AWR is capable of arbitrary waveforms as shown by this composite waveform made up of arbitrary waveform parts: (1) triangular wave (2) linear chirp (3) ramp and (4) composite waveform made up by direct addition of 3 sine waves of equal amplitude but different frequencies (5, 7 and 12 kHz). The drive waveform shape is validated by the internal current monitor of the AE Techron 7224.

**Figure 7:**
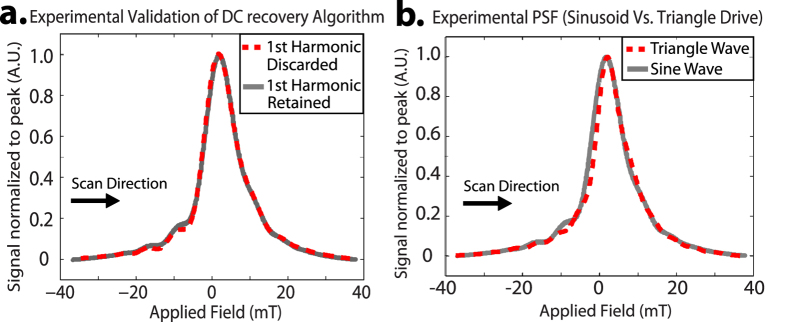
(**a**) First experimental validation of the DC recovery algorithm used in x-space reconstruction. DC recovery of the discarded first harmonic information produces a nearly identical PSF to a PSF where the analog received signal at the first harmonic was retained. (**b**) Experimentally reconstructed PSF for Triangle and Sine drive wave showing only minor differences. This is expected because a triangle and sine wave are similar, and the x-space theory in 1D shows that various trajectories with similar spatial sampling densities should yield the same reconstructed results, excepting variable impacts of magnetic relaxation. Importantly, this shows the triangle wave maintains conventional MPI performance while obviating velocity compensation which is a major reconstruction step.

While the wide, continuous frequency range of the AWR is useful, the most unique feature of the AWR is the capability to generate and apply arbitrary drive waveforms. Such waveforms have never been experimentally evaluated in an MPI context and may contain much potential since recent pioneering work by Vogel *et al*.^45^ has increasingly pointed to the benefits of non-sinusoidal drive waveforms, in particular triangular waves. Since it is technically challenging and expensive to implement arbitrary waveforms on an imaging scanner, our novel AWR is an indispensable tool to evaluate the pros and cons of a proposed arbitrary drive waveform before implementing on a small-animal or human-scale. For example, in our comparison of a triangle drive waveform to an equivalent sine drive waveform (Fig. 7), we show that triangular drive waveforms are able to maintain similar MPI performance to conventional sinusoids while significantly reducing reconstruction computational load. In addition, as shown by the Arbitrary Waveform in Fig. 6, the differing responses of Resovist and Senior Scientific 32 nm SPIOs to the different arbitrary waveforms suggests potential of our device to optimize waveform shape, amongst other parameters, to improve contrast between tracers for color MPI applications first pioneered by Rahmer *et al*.^15^. The capability of the AWR is not limited to classic waveforms such as triangle and square waves, and more complex waveforms constructed by summing multiple basic or primitive waveforms are also possible as shown in Fig. 6. While the full optimization of arbitrary waveforms for MPI performance is outside the scope of this paper, we have demonstrated the hardware capability to conduct this study.

## Conclusion

Recent work from Kuhlmann *et al*. and Vogel *et al*.^17^,^29^,^45^ have pointed towards the importance of frequency-flexible and non-sinusoidal drive waveforms for improved MPI spatial resolution and SPIO relaxation contrast. As a result, it is of interest for Magnetic Particle Spectrometers (MPS) to optimize the drive waveform in addition to its basic role of particle characterization. Unfortunately, to date, there has been no MPS hardware capable of the wide-range frequency-flexibility or the arbitrary drive waveforms required for a comprehensive optimization. In response to this, we have designed a novel tabletop magnetic particle spectrometer and relaxometer (AWR) with high-throughput capability, unprecedented frequency-agility (DC – 400 kHz) and unique capability for arbitrary drive waveforms. This greatly expands the parameter space available for drive waveform optimization. We believe the unprecedented flexibility of the AWR will be of interest to MPI system designers and nanoparticle experts seeking to optimize and tailor MPI drive waveforms to the SPIO tracers for improved spatial resolution, signal strength and microenvironment contrast.

## Additional Information

**How to cite this article**: Tay, Z. W. *et al*. A High-Throughput, Arbitrary-Waveform, MPI Spectrometer and Relaxometer for Comprehensive Magnetic Particle Optimization and Characterization. *Sci. Rep.* **6**, 34180; doi: 10.1038/srep34180 (2016).
